# A new species of the web-inhabiting bug genus *Heissophila* Schuh (Hemiptera, Plokiophilidae) from China, with notes on host associations for the genus

**DOI:** 10.3897/zookeys.1296.200871

**Published:** 2026-10-27

**Authors:** Haoyang Xiong, Zhuo Chen, Hu Li, Wanzhi Cai

**Affiliations:** 1 State Key Laboratory of Agricultural and Forestry Biosecurity, MARA Key Lab of Pest Monitoring and Green Management, College of Plant Protection, China Agricultural University, Beijing 100193, China State Key Laboratory of Agricultural and Forestry Biosecurity, MARA Key Lab of Pest Monitoring and Green Management, College of Plant Protection, China Agricultural University Beijing China

**Keywords:** Heissophilinae, Heissophilini, identification key, morphology, Oriental region, taxonomy

## Abstract

A new species of web-loving bug, *Heissophila sinica***sp. nov**. (Hemiptera, Heteroptera, Plokiophilidae, Heissophilinae), is described from Fujian and Guangdong, China. The species was found inhabiting the webs of *Orientothele* sp. and *Vacrothele* sp. (Araneae, Macrothelidae). It represents the second described species of the genus *Heissophila* Schuh, 2006 and expands the distributional records of the genus in the Oriental region. The new species can be distinguished from its only known congener by its smaller body size, predominantly brachypterous form, veinless hemelytral membrane, and corial glands with distinct morphology restricted to the hemelytra. The generic diagnosis of *Heissophila* is revised, a key to species of the genus and a new distribution map are provided. Additionally, the habitat, host spiders, and biological characteristics of the new species are briefly documented.

## Introduction

The family Plokiophilidae comprises a group of small predatory true bugs, with a body length ranging from 1.10 to 3.00 mm (China and Myers 1929; China 1953; Carayon 1974). A total of nine genera and 24 species have been recorded (including one Baltic amber fossil genus and one fossil species) (Popov 2008; Xiong et al. 2025, 2026). They are placed in the cimicomorphan superfamily Cimicoidea and are hypothesized to be the sister group to the Cimicidae, Curaliidae, and Polyctenidae (Schuh et al. 2009; Henry 2017; Weirauch et al. 2018; Schuh and Weirauch 2020). Species of this family exhibit unique behavioral habits and are rarely collected, with most members of Plokiophilidae mainly inhabiting the webs of spiders or embiopterans, completing their entire life cycles on the host webs, and their food sources encompassing the hosts’ food, eggs, juveniles, weakened adults, as well as other small arthropods attached to the host webs (Carayon 1974; Büscher et al. 2023). A few additional species have been discovered in flight interception traps, Malaise traps, soil samples and leaf litter samples (Štys 1967, 1991; Carpintero and Dellapé 2005; Štys and Baňař 2016; Luo et al. 2021).

China and Myers (1929) discovered *Arachnophila cubana*, the first species of the Plokiophilidae, on the webs of *Diplura macrura* (Koch, 1841) in Cuba, and tentatively placed it within the family Microphysidae. Due to the homonymy conflict between *Arachnophila* China & Myers, 1929 and *Arachnophila* Salvadori, 1874 (Aves), China (1953) revised the former generic name to *Plokiophila* China, 1953, established the subfamily Plokiophilinae, and described a new genus and species collected from the webs of embiopterans on the island of Trinidad. Carayon (1961) recognized Plokiophilidae as a family distinct from Microphysidae and divided the family into the subfamilies Plokiophilinae and Embiophilinae based on host differences. Štys (1962, 1967) supported this taxonomic change, provided a key to Plokiophilidae, and described one new genus and species from Brazil. Carayon (1974) presented more detailed morphological taxonomic characters for the family and the two previously proposed subfamilies, and described one new genus and seven new species from continental Africa and Brazil. In the subsequent three decades, three new species of Plokiophilidae were described (Štys 1991; Eberhard et al. 1993; Carpintero and Dellapé 2005). Schuh (2006) described a new genus and species, *Heissophila macrotheleae*, from Thailand, which differs from other Plokiophilidae in head structure, pronotum, corial glands, and male and female genitalic structures, and its placement at the subfamily level was tentatively treated as incertae sedis due to the lack of additional material. Schuh et al. (2015), based on male and female genitalic structures from additional specimens, downgraded Embiophilinae to tribal rank within the subfamily Plokiophilinae, established the new subfamily Heissophilinae and the new tribe Lipokophilini. Since then, all taxonomic studies on Plokiophilinae (Štys and Baňař 2016; Henry 2020; Luo et al. 2021; Xiong et al. 2025, 2026) have adopted the classification system proposed by Schuh et al. (2015).

The genus *Heissophila* Schuh, 2006 was originally established by Schuh (2006) based on specimens collected from Chiang Mai, Thailand, with *Heissophila macrotheleae* Schuh, 2006 as its only described species. However, since traumatic insemination via copulatory tubes or puncture of the abdominal wall is absent in this group, its subfamilial placement could not be determined at that time. Schuh et al. (2015) established the subfamily Heissophilinae based on the structure of both male and female genitalia and placed the genus within it. This genus is distributed in East and Southeast Asia, with recorded occurrences in Thailand, Indonesia, and China (Schuh 2006; Yin et al. 2023).

During our recent field investigations in Fujian, southern China, we collected a number of specimens of a plokiophilid species from the webs of a spider belonging to the genus *Orientothele* Mirza, Sanap & Kunte, 2017 (Araneae, Macrothelidae) (Mirza et al. 2017). In addition, we received a series of specimens collected from the webs of spiders of the genus *Vacrothele* Tang & Yang, 2022 (Araneae, Macrothelidae) in Guangdong Province, southern China (Tang et al. 2022). Further examination revealed that these specimens all represent a new species of *Heissophila*. This new species represents the second member of the genus *Heissophila*. New distribution records for species of *Heissophila* are documented, the generic diagnostic characteristics of the genus are discussed and revised, and the ecological characteristics of the new species are briefly reported.

## Material and methods

Specimens examined in this study were deposited in the Entomological Museum of China Agricultural University, Beijing, China (**CAU**).

External and genital structures were examined using a Nikon SMZ745 stereoscopic microscope. Measurements (in mm) were taken using a Keyence large depth-of-field microscope (VHX-X1) with Adobe Photoshop 2024. Male genitalia and female abdomens were macerated in 10% potassium hydroxide solution (KOH) at 60 °C for 3 h. Photographs were taken using the Keyence large depth-of-field microscope (VHX-X1). Ecological photos were taken with a Nikon D5 camera with a LAOWA 100 mm f/2.8 lens. Scanning electron micrographs were prepared using a Regulus 8100 Scanning Electron Microscope at State Key Laboratory for Biology of Plant Diseases and Insect Pests, Institute of Plant Protection, Chinese Academy of Agricultural Sciences, Beijing, China. Figure plates were prepared using Adobe Photoshop 2024. The distribution map was prepared using QGIS Desktop 3.34.11.

The classification system of Plokiophilidae follows Schuh et al. (2015). Morphological terminology mainly follows China and Myers (1929) and Schuh et al. (2015). In terms of measurements, length of body is defined as the straight-line distance from the anterior tip of the head to the posterior end of the abdomen; maximum width of pronotum is defined as the distance between the two posterolateral angles of the pronotum; and maximum length of pronotum is defined as the perpendicular distance from the anterior margin of the pronotum to the line connecting the posterolateral angles.

Abbreviations used in the text and figures are as follows:

**aed** aedeagus;

**cgs** corial glands;

**Cu** cubitus;

**gps** gland pores;

**lp** left paramere;

**M** media;

**o** ostiole;

**og** ostiolar groove;

**pe** parempodia;

**R** radius;

**rp** right paramere;

**Sc** subcostal;

**se** seta;

**sv** secondary vein;

**ts1** first segment of tarsus;

**ts2** second segment of tarsus;

**ts3** third segment of tarsus;

**1A** first anal vein.

## Taxonomy

### Genus Heissophila

Taxon classificationAnimaliaHemipteraChlamydiaceae

Schuh, 2006

33C60A03-7B94-5DB9-9F97-EEC95FF3463C

Heissophila Schuh, 2006: 637. Type species by original designation: Heissophila macrotheleae Schuh, 2006.

#### Diagnosis.

*Heissophila* can be distinguished from other genera of Plokiophilidae by the following combination of character states: head lacking elongate neck behind eyes; fossula spongiosa absent on all legs; tarsi 3-segmented; pygophore broader at base than apex; parameres short, thick and right-angled; endosoma in repose in the form of a tube; female abdomen with presence of ovipositor valves.

#### Diversity and distribution.

The genus previously comprised only one species, with a distributional range covering China, Indonesia and Thailand (Schuh 2006; Yin et al. 2023). The new species described here is distributed in southeastern coastal regions of China.

### Heissophila sinica sp. nov.

Taxon classificationAnimaliaHemipteraChlamydiaceae

C9E39337-437A-564A-AF45-424CB2558DA3

https://zoobank.org/5733EE04-B818-4352-B646-97E9A307A818

Figs 1, 2, 3, 4, 5

#### Chinese vernacular name.

中华海丝蝽.

#### Type material.

***Holotype***: • ♂, China, Fujian, Fuzhou Dist. [闽侯县], Minhou, Qishan National Forest Park [旗山国家森林公园], 25.9916°N, 119.1913°E, c. 77 m, 28.ix.2025, leg. Haoyang Xiong, Zhuo Chen & Feiyi Xin (CAU, accession number: CAUFJ-PLO1). ***Paratypes***: • 6♂♂, 6♀♀ and 4 nymphs, same data as for holotype (CAU, accession number: CAUFJ-PLO1); • 2♂♂, 3♀♀, China, Fujian, Nanping Dist. [延平区], Yanping, Fujian Agriculture and Forestry University [福建农林大学], 26.5591°N, 118.1105°E, c. 115 m, 11.iv.2026, leg. Feiyi Xin (CAU, accession number: CAUFJ-PLO2); • 1♂, 2♀♀, China, Guangdong, Guangzhou Dist. [天河区], Tianhe, Longdong Reservoir [龙洞水库], 23.2266°N, 113.3861°E, c. 80 m, 1.ii.2026, leg. Nuoshen Feng (CAU, accession number: CAUGD-PLO1); • 2♂♂, 2♀♀, China, Guangdong, Guangzhou Dist. [天河区], Tianhe, Longdong Reservoir [龙洞水库], 23.2266°N, 113.3861°E, c. 80 m, 1.ii.2026, leg. Nuoshen Feng (SCAU, accession number: SCAUGD-PLO1); • 1♂, 1♀, China, Guangdong, Jiangmen Dist. [新会区], Xinhui, Daling [大岭], 22.3730°N, 113.1317°E, c. 208 m, 20.i.2026, leg. Nuoshen Feng (CAU, accession number: CAUGD-PLO2); • 3♂♂, 2♀♀, China, Guangdong, Jiangmen Dist. [新会区], Xinhui, Daling [大岭], 22.3730°N, 113.1317°E, c. 208 m, 20.i.2026, leg. Nuoshen Feng (SCAU, accession number: SCAUGD-PLO2).

#### Diagnosis.

This species is recognized within the genus by the following combination of character states: brachypterous predominantly; body length 1.1–1.4 mm; antennal segments II and III subequal in length; tarsomere II distinctly shorter than tarsomere III; membrane veinless; corial glands present only on the corial area of the hemelytron; corial glands circular, droplet-shaped, convex throughout, with the gland pore located on the convexity.

#### Description.

**Figure 1.**
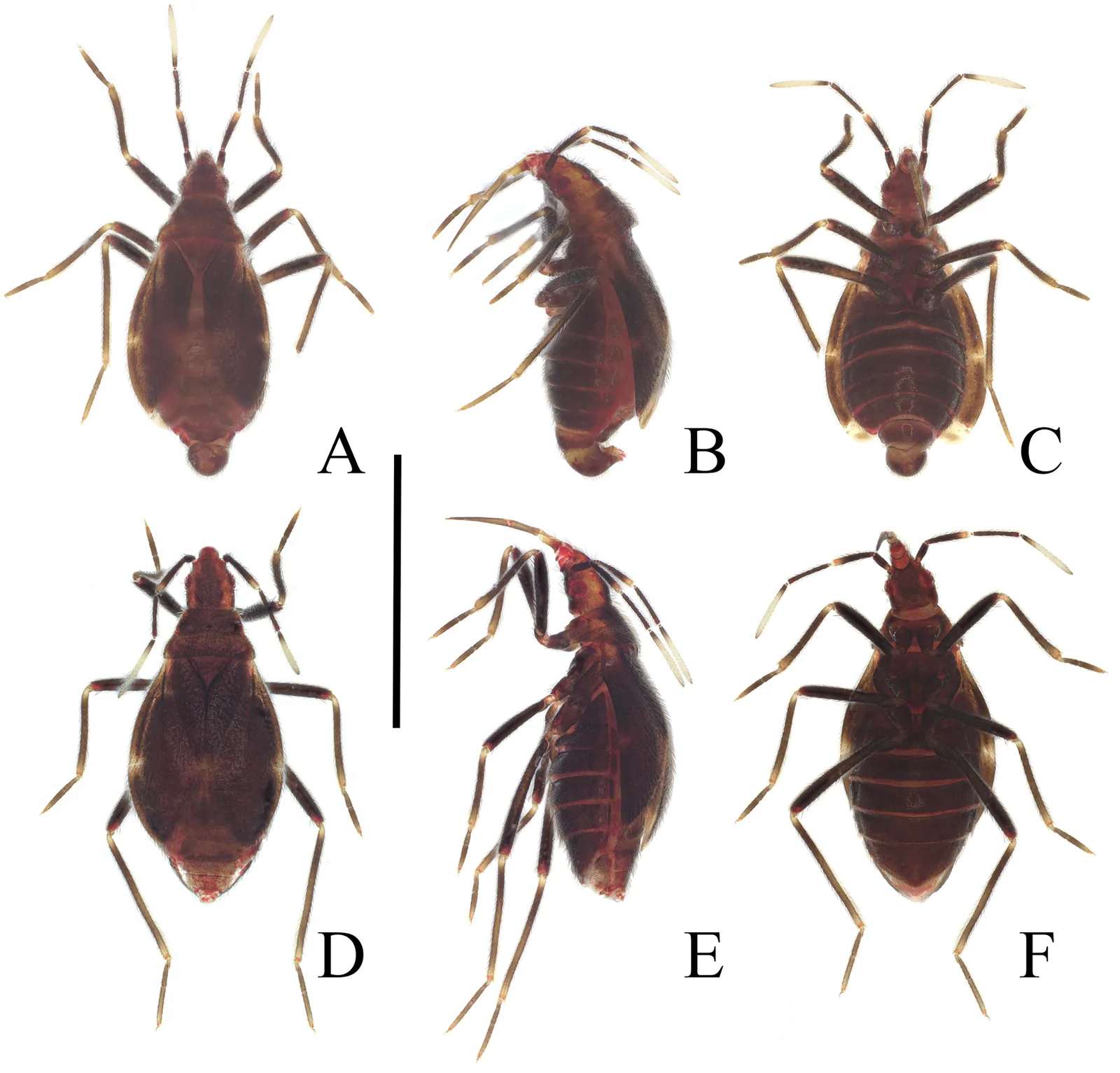
*Heissophila sinica* sp. nov., brachypterous habitus. **A**. Male, holotype, dorsal view; **B**. Same, lateral view; **C**. Same, ventral view; **D**. Female, paratype, dorsal view; **E**. Same, lateral view; **F**. Same, ventral view. Scale bar: 1 mm.

**Figure 2.**
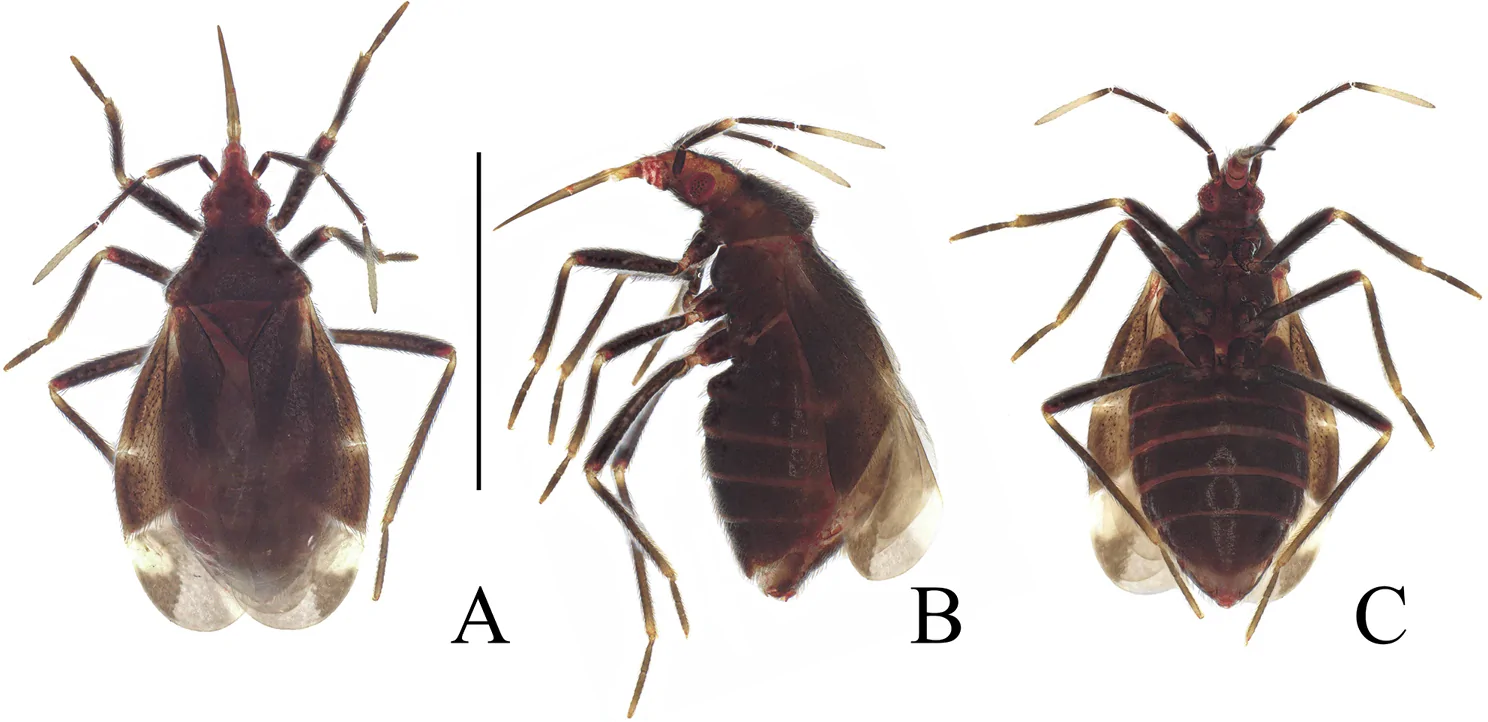
*Heissophila sinica* sp. nov., macropterous habitus. **A**. Female, paratype, dorsal view; **B**. Same, lateral view; **C**. Same, ventral view. Scale bar: 1 mm.

**Figure 3.**
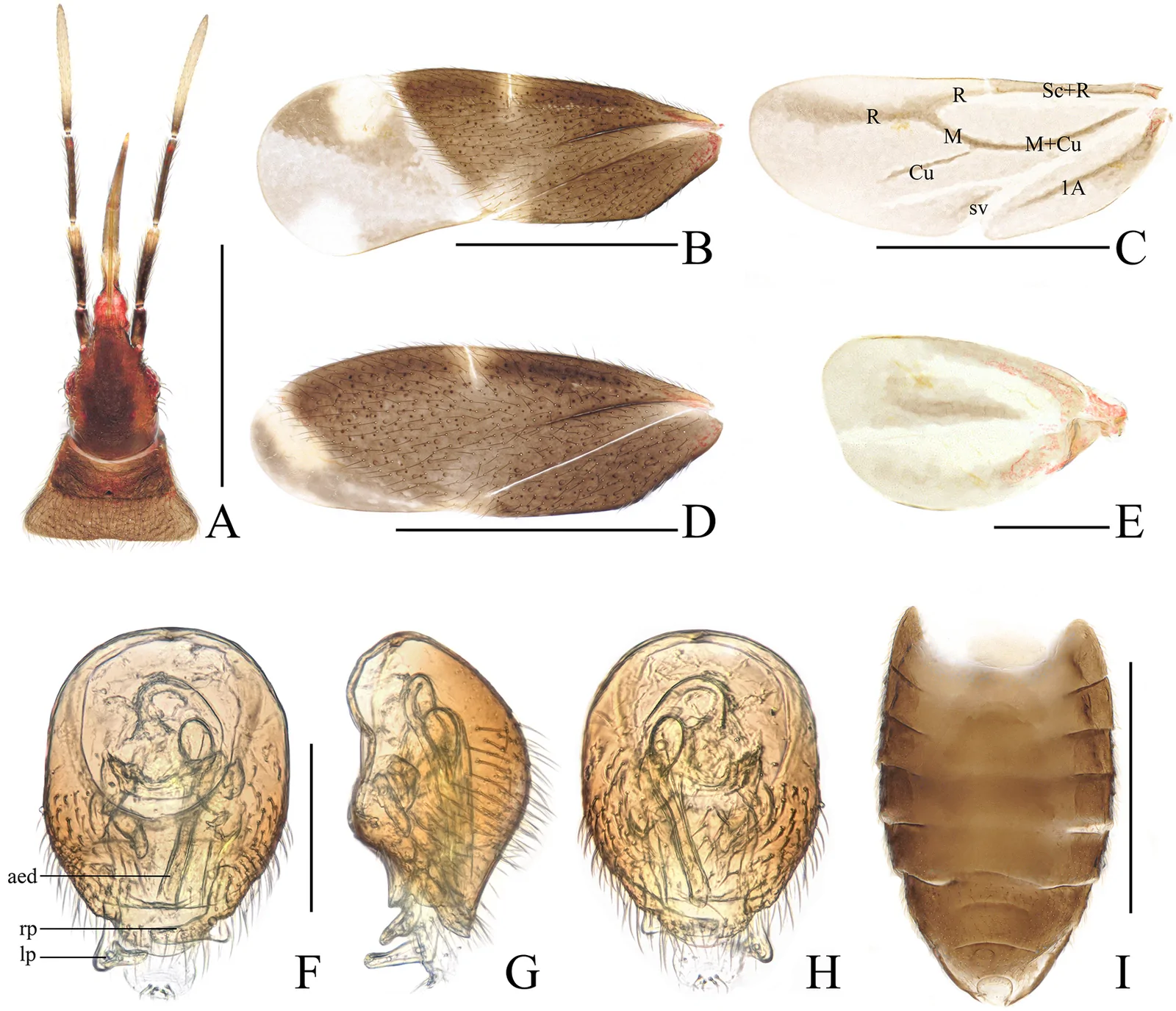
*Heissophila sinica* sp. nov., diagnostic morphological characters. **A**. Head and pronotum, brachypterous, dorsal view; **B**. Hemelytron, macropterous, dorsal view; **C**. Hind wing, macropterous, dorsal view; **D**. Hemelytron, brachypterous, dorsal view; **E**. Hind wing, brachypterous, dorsal view; **F**. Male genitalia, dorsal view; **G**. Same, lateral view; **H**. Same, ventral view; **I**. Female abdomen, dorsal view. Abbreviations: aed = aedeagus, lp = left paramere, rp = right paramere. Scale bars: 0.5 mm (**A–D, I)**; 0.1 mm (**E–H**).

Brachypterous male and female (Fig. 1), macropterous female (Fig. 2). ***Coloration***. Generally dark brown. Reddish-brown longitudinal stripes on both sides of vertex; labrum and eyes reddish brown (Fig. 3A). Antennal segment 2 apex and segment 4 except base pale, remaining segments dark. Rostrum yellowish-brown, second segment paler. Legs: tibiae and tarsi brown, tibiae pale at both ends. Brachypterous hemelytra (Fig. 3D): corium base, clavus apex and cuneal fracture pale, membrane brown with pale patches at base. Macropterous with ocelli reddish brown; membrane bearing a single pale patch inside apex (Fig. 3B); hind wings brown, veins slightly darker (Fig. 3C); other characters same as brachypterous.

**Figure 4.**
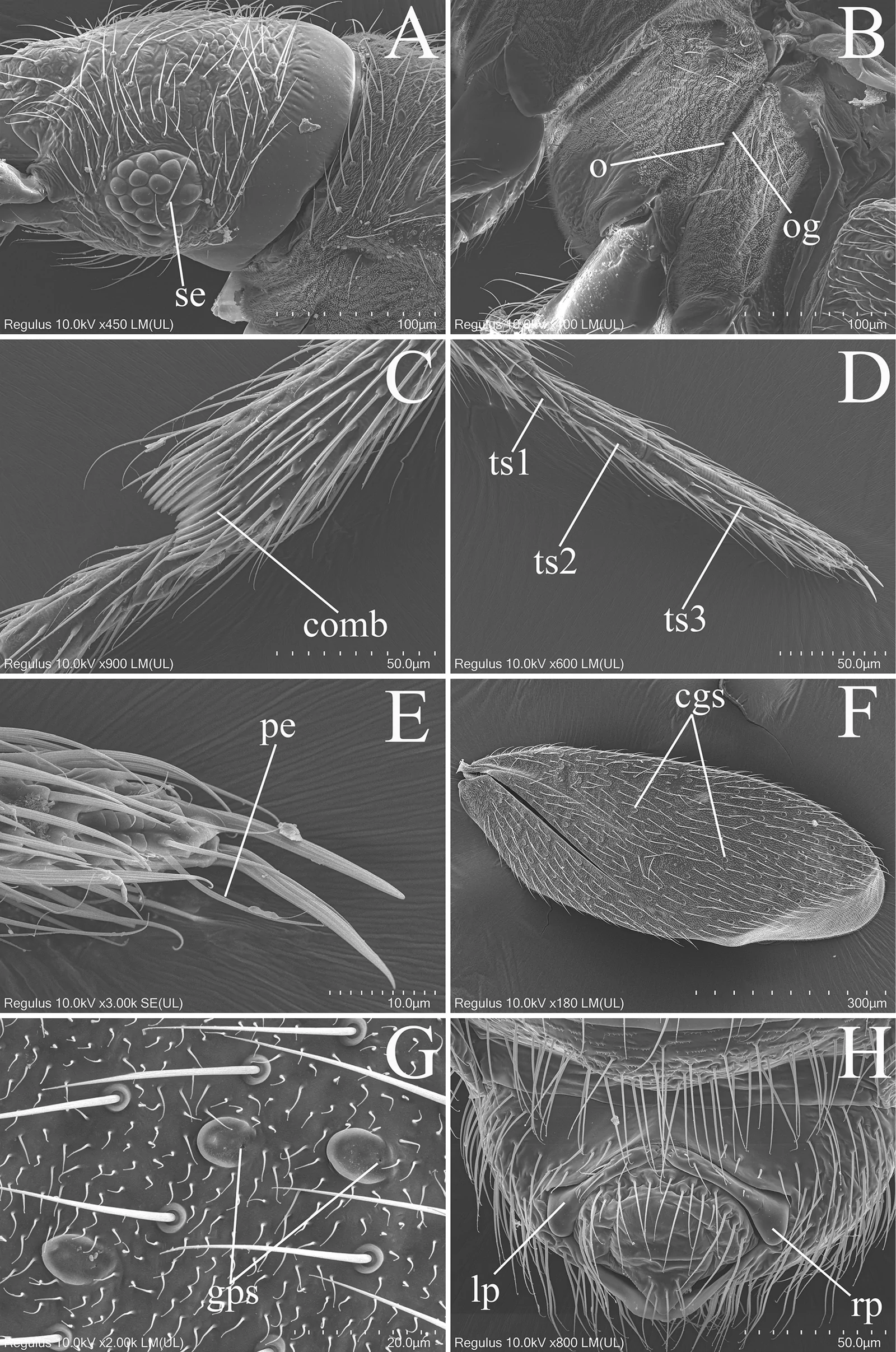
Scanning electron micrographs of *Heissophila sinica* sp. nov. **A**. Male head, brachypterous, lateral view, showing setae inside compound eye; **B**. Thorax, lateral view, showing ostiole and ostiolar groove; **C**. Apex of fore tibia, lateral view, showing comb; **D**. Fore tarsus, lateral view; **E**. Claws of fore leg, showing parempodia; **F**. Hemelytron, brachypterous, dorsal view, showing distribution area of corial glands; **G**. Corial glands, dorsal view, showing gland pores; **H**. Male genitalia, brachypterous, dorsal view, showing paramere. Abbreviations: cgs = corial glands, gps = gland pores, lp = left paramere, o = ostiole, og = ostiolar groove, pe = parempodia, rp = right paramere, se = seta, ts1 = first segment of tarsus, ts2 = second segment of tarsus, ts3 = third segment of tarsus.

***Vestiture***. Body glossy, covered with short, semi-erect, simple setae. Head with long, semi-erect setae; occiput glabrous; antennal segment 1 with sparse setae, segments 2–4 dense; rostrum segment 1 to base of segment 4 with sparse setae; compound eye with two long setae between ommatidia (Fig. 4A). Thorax with short, dense pubescence; ventral side denser than dorsal side; pronotum with long, semi-erect setae; calli sparsely vestited; scutellum with sparse semi-erect setae. Legs densely vestited; coxae sparse; front tibia apex with a tuft of hairs representing incipient grasping organ and comb for antennal cleaning (Fig. 4C). Hemelytra densely covered with pubescence; corium and clavus with uniformly distributed long, semi-prostrate setae (inclination angle of semi-erect setae on body surface ranges from 30° to 60°, while included angle between setae on wings and wing surface measures less than 30°); corium ventral outer margin with a row of setae. Abdomen with long, semi-erect setae except abdominal terga I–V; male genital segment with semi-erect setae; dorsal side sparse; parameres apex with sparse setae (Fig. 4H); macropterous same as brachypterous.

**Figure 5.**
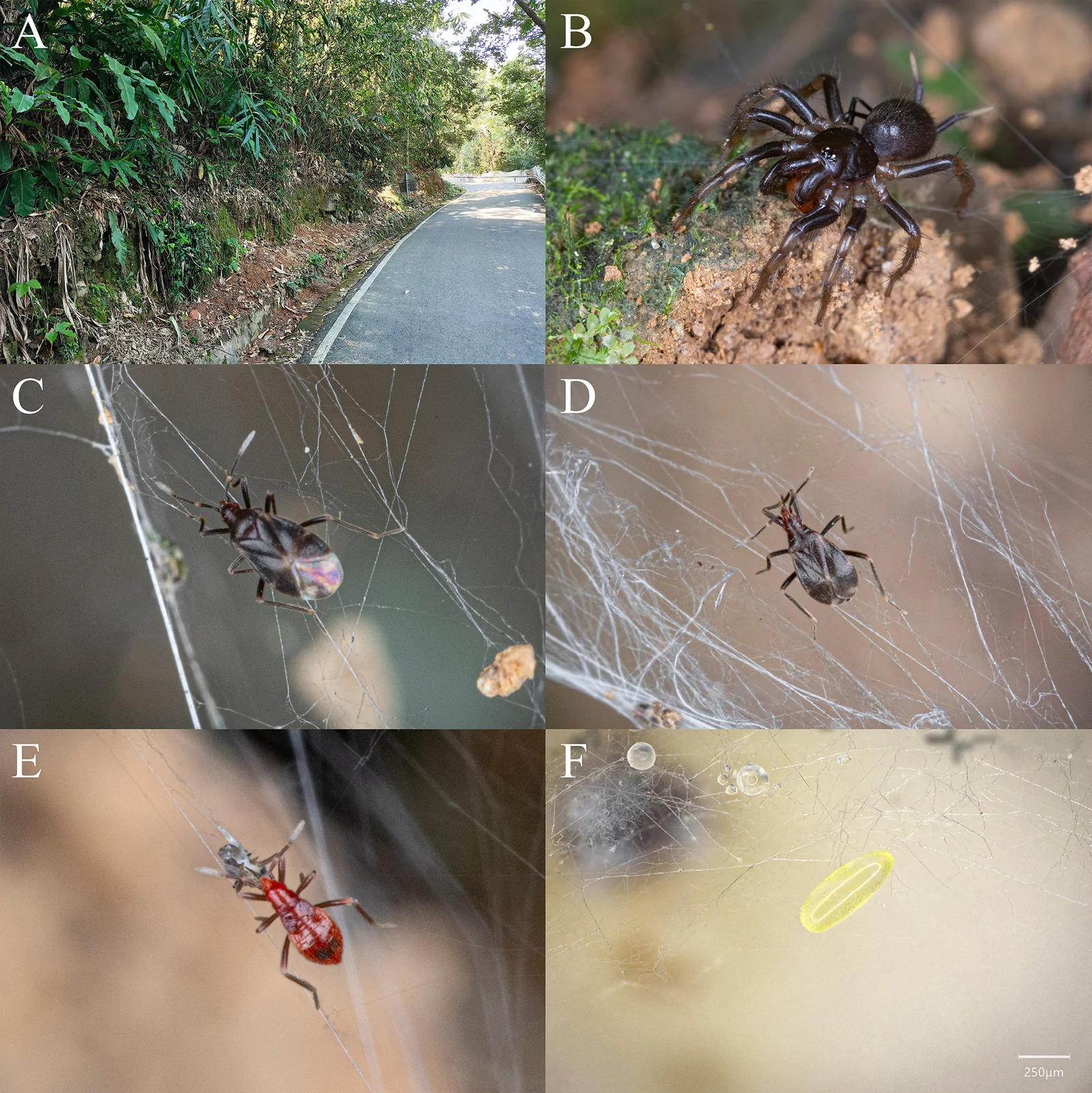
Habitats, spider host and living individuals of *Heissophila sinica* sp. nov. **A**. Habitat in type locality; **B**. Spider host, *Orientothele* sp.; **C**. Macropterous female adult of *Heissophila sinica* sp. nov., dorsal view; **D**. Brachypterous male adult of *Heissophila sinica* sp. nov., dorsal view; **E**. Nymph of *Heissophila sinica* sp. nov.; **F**. Egg of *Heissophila sinica* sp. nov.

***Structure*. *Head*** (Fig. 3A) nearly cylindrical, distinctly narrowed towards apex in dorsal view, width slightly exceeding length; tylus broad, widening towards truncated apex; eyes small, remote from prothorax; brachypterous without ocelli, macropterous ocelli small, distance between ocellus center and eye less than between two ocellus centers; antennae slender, segment I stouter and shorter than remaining segments, length three times width; antennal segment length ratio I:II:III:IV = 1:2.2:2.5:3.8; female = 1:2.1:2.4:3.4 (see Table 1); labium slender, apex extending to mid coxae, segment I short and broad; labial segment length ratio I:II:III:IV = 1:1.5:2.3:4.5.

**Table 1.** Measurements (in mm) of *Heissophila sinica* sp. nov.

**Body part**	**Brachypterous male (holotype)**	**Brachypterous male (paratypes, *N* = 5)**	**Brachypterous female (paratypes, *N* = 5)**	**Macropterous female (paratypes, *N* = 1)**
Length of body	1.20	1.18–1.24	1.29–1.36	1.36
Length of head	0.19	0.18–0.19	0.21–0.22	0.23
Width across eyes	0.17	0.17–0.19	0.18–0.2	0.21
Interocular space	0.12	0.11–0.12	0.12–0.14	0.14
Length of antennal segment I	0.07	0.07	0.07–0.08	0.08
Length of antennal segment II	0.17	0.16–0.18	0.16–0.18	0.17
Length of antennal segment III	0.18	0.17–0.18	0.17–0.19	0.19
Length of antennal segment IV	0.27	0.25–0.28	0.26–0.27	0.27
Length of labial segment I	0.05	0.05	0.05	0.05
Length of labial segment II	0.09	0.09–0.1	0.09	0.1
Length of labial segment III	0.12	0.12–0.13	0.11–0.13	0.13
Length of labial segment IV	0.24	0.24–0.26	0.25–0.27	0.25
Maximum length of pronotum	0.17	0.16–0.19	0.18–0.21	0.22
Maximum width of pronotum	0.30	0.30–0.33	0.33–0.37	0.44
Length of fore femur	0.27	0.27–0.30	0.26–0.32	0.31
Length of fore tibia	0.27	0.27–0.29	0.29–0.31	0.30
Length of fore tarsus	0.18	0.18–0.19	0.17–0.19	0.19
Length of foreleg tarsomere II	0.06	0.06–0.07	0.05–0.06	0.05
Length of foreleg tarsomere III	0.11	0.1–0.11	0.11	0.11
Length of mid femur	0.32	0.30–0.33	0.31–0.33	0.33
Length of mid tibia	0.32	0.32–0.35	0.36–0.37	0.37
Length of mid tarsus	0.18	0.17–0.19	0.19	0.19
Length of hind femur	0.44	0.42–0.47	0.44–0.5	0.49
Length of hind tibia	0.47	0.46–0.5	0.47–0.49	0.52
Length of hind tarsus	0.23	0.23–0.26	0.26	0.27
Length of hemelytron	0.84	0.81–0.89	0.84–0.87	1.04
Length of abdomen	0.76	0.73–0.8	0.74–0.79	0.82
Maximum width of abdomen	0.48	0.46–0.48	0.47–0.5	0.55

***Pronotum*** (Fig. 3A) trapezoidal, maximum width slightly less than twice maximum length; with narrow collar; brachypterous lateral margins nearly straight, macropterous lateral margins slightly concave medially; posterior margin slightly concave medially, elevated in lateral view; posterior angles obtuse; calli small, ovate, mutually remote, adjacent to lateral margins. Scutellum with basal width slightly greater than length. Ostioles visible on anterior margin of metepisternum; ostiolar groove and evaporatorium limited in extent (Fig. 4B).

***Legs*** relatively slender; coxae cylindrical. Femora slightly thickened at middle, spineless; fore tibia with cleaning comb on medial surface at apex (Fig. 4C); no fossula spongiosa. Tibiae slightly thickened towards apex; tarsi relatively stout, moderately long, 3-segmented, segment 1 very short, segment 2 shorter than segment 3 (Fig. 4D). Claws elongate, slender, tapering to sharply pointed apex; inner claw slightly longer than exterior; parempodia (Fig. 4E) well developed, setiform, subequal in length; of similar structure on all 3 pairs of legs.

***Macropterous hemelytra*** (Fig. 3B) relatively broad, reaching abdominal apex; corial costal margin weakly sinuous, coriomembranal juncture nearly straight, well defined; with costal fracture, located at two-thirds distance from corial base, length about one-eighth that of corial costal margin; corial process present sublaterally on membrane at coriomembranal juncture; without cuneus; membrane veinless. Corial glands numerous but small, evenly distributed over entire corium and clavus; corial glands circular, droplet-shaped, convex from base to apex, with 1 gland pore at base (Fig. 4G). Hind wing venation as shown in Fig. 3C; Cu with distal free branch.

***Brachypterous hemelytra*** (Figs 3D, 4F) relatively short, covering only anterior five abdominal segments; corium elevated, elytron-like; corial costal margin slightly curved, smooth; coriomembranal juncture curved outward, indistinct; with costal fracture, located at two-thirds distance from corial base, length about one-twelfth that of corial costal margin; membrane small, veinless. Hind wings (Fig. 3E) reduced, triangular, veinless. Other characters same as macropterous.

***Abdomen*** (Fig. 3I) oval, length about 1.7 times maximum width; sterna entire, ventrolateral tergites fused with sterna but demarcated by sublateral impression; sternum of segment 2 strongly convex medially; terga widely separated from dorsal laterotergites by broad membranous area; abdomen expandable into ellipsoid shape after feeding.

***Male genitalia*** (Figs 3F–H, 4H): pygophore short and broad, telescoped within abdominal segments 7 and 8, opening dorsad; parameres arising laterally from a semicircular excavation, without a dorsal thumb-like projection, closely appressed to dorsal apex of pygophore opening, directed toward opposite side; endosoma tubular, membranous; parameres bilaterally symmetrical, narrow at base, right-angled at middle, broad and flattened at apex, with a few short setae on apicodorsal margin.

***Female genitalia***: broad, saccate.

#### Etymology.

The specific epithet refers to China, the country of the type locality of the new species.

#### Distribution.

**Figure 6.**
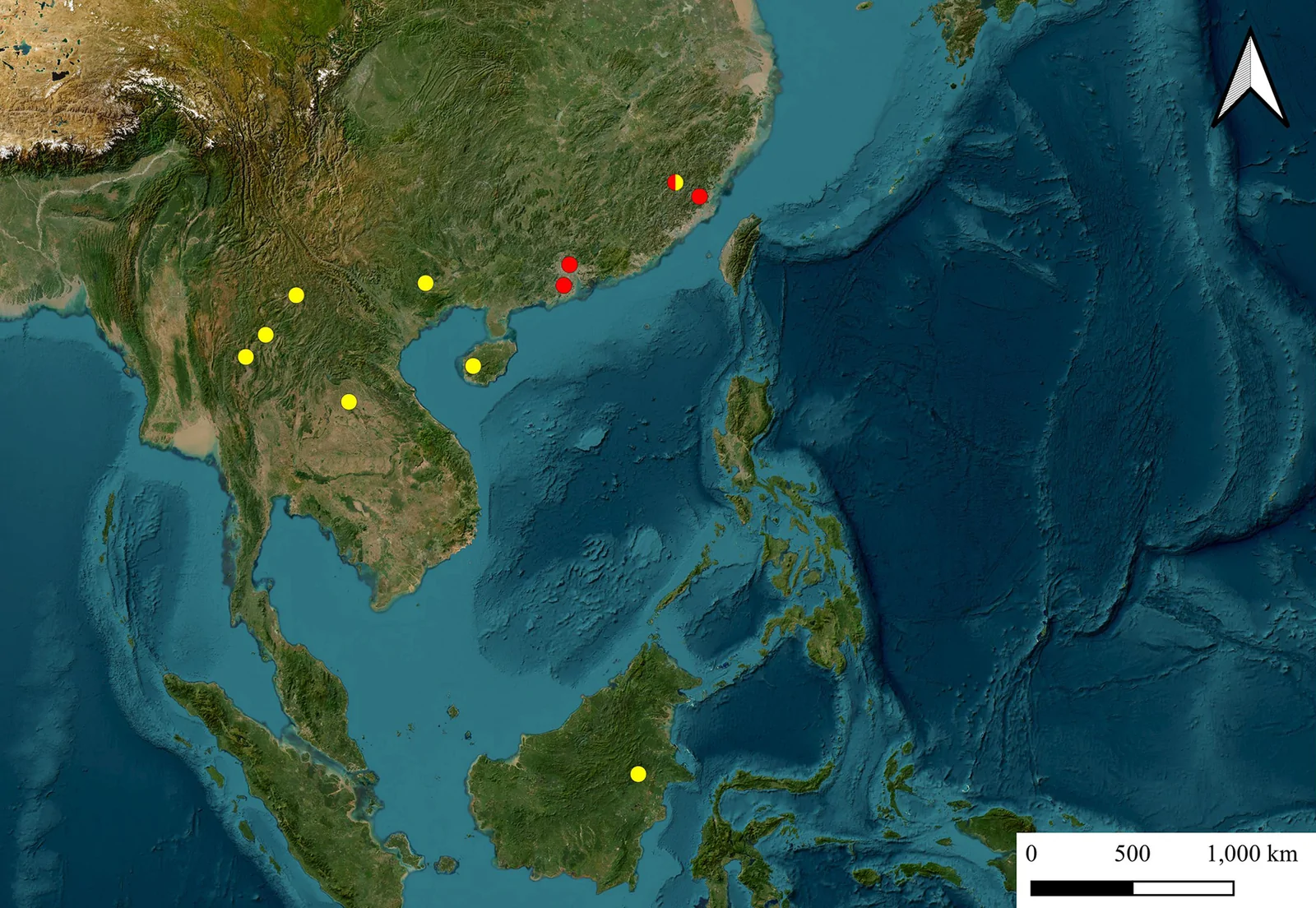
Known distribution of *Heissophila*: yellow dot = *H. macrotheleae*; red dot and star = *H. sinica* sp. nov.

China (Fujian; Guangdong) (Fig. 6).

##### Key to species of *Heissophila*

**Table:** 

1	Brachypterous predominantly; body small, length less than 1.5 mm; antennal segments II and III subequal in length; tarsomere II distinctly shorter than tarsomere III; membrane veinless; corial glands present only on the coriaceous portion of the hemelytra; corial glands circular, droplet-shaped, convex throughout, with the gland pore located on the convexity	***H. sinica* sp. nov**.
–	Macropterous; body large, length greater than 2 mm; antennal segment II longer than segment III; tarsomere II subequal to tarsomere III in length; membrane with four veins; corial glands distributed on the coriaceous portion of the hemelytra, pronotum and antennal segment II; corial glands with a circular convexity in the center and a depression around, gland pore located within the depression	***H. macrotheleae***

## Discussion

### Comparative notes

Schuh et al. (2015) divided the family Plokiophilidae into Plokiophilinae and Heissophilinae based on the presence or absence of traumatic insemination structures in the male and female genitalia. Heissophilinae comprises the genera *Heissophila* Schuh, 2006, *Monteithophila* Schuh, Štys & Cassis, 2015, and *Pavlostysia* Popov, 2008 (a fossil genus from Baltic amber). Observations under light microscopy reveal that the male of *H. sinica* sp. nov. possesses an endosoma in the form of a tube, and the female has well-developed ovipositor valves internally, with no traumatic insemination structures present. Accordingly, this new species is assigned to Heissophilinae.

Regarding generic assignment, morphological evidence indicates that *H. sinica* sp. nov. can be distinguished from *Monteithophila* and *Pavlostysia* by the following combination of characters: without neck behind eyes, cuneus absent, pronotum not strongly elevated in lateral view, and tarsal claws subequal (Popov 2008; Schuh et al. 2015). According to Schuh (2006), *Heissophila* is characterized by: 3-segmented tarsi; four veins (dead) in the membrane; short broad pygophore; short, stout, right-angled parameres; endosoma in repose in the form of a tube with many internal spicules; presence of ovipositor valves within the female abdomen; and distribution in the Old World. *Heissophila sinica* sp. nov. matches all the original diagnostic characters of *Heissophila* except for the absence of veins in the hemelytral membrane. Considering that the presence or absence of veins and the formation of cells in the membrane vary among species in both *Plokiophiloides*Carayon 1974 and *Neoplokioides* Štys & Baňař, 2016, we refrain from erecting a new genus based solely on this venational character following conservative taxonomic practice. Instead, we assign *H. sinica* sp. nov. to *Heissophila* and provide a revised generic diagnosis. The revised diagnostic characters of *Heissophila* are as follows: head lacking an elongate neck behind eyes; fossula spongiosa absent on all legs; tarsi 3-segmented; pygophore broader at base than apex; parameres short, thick and right-angled; endosoma in repose in the form of a tube; and female abdomen with presence of ovipositor valves. The specific taxonomic placement of this species requires further investigation based on additional specimen material.

Previously, the genus *Heissophila* included only *H. macrotheleae*. In comparison, *H. sinica* sp. nov. exhibits the following differences in addition to the absence of veins in the hemelytral membrane: it is predominantly brachypterous, whereas no brachypterous individuals have been reported for *H. macrotheleae*; it is smaller in size, with most individuals less than 1.5 mm in body length, while all specimens of *H. macrotheleae* exceed 2 mm; the second antennal segment is subequal to the third, whereas in the known species the second segment is distinctly longer than the third; tarsomere II is shorter than tarsomere III, while they are nearly equal in the known species; corial glands are restricted to the hemelytra, whereas in *H. macrotheleae* they also occur on the pronotum and the second antennal segment; corial glands are circular, droplet-shaped, convex from base to apex, with a gland pore at the base. Additionally, the male terminalia of the two species show distinct differences. In *H. sinica* sp. nov., the pygophore is subglobose in lateral view and lacks the dorsal thumb-like projection; the apical portion of the parameres is nearly straight. In contrast, the pygophore of *H. macrotheleae* is subtriangular in lateral view, with a dorsal thumb-like projection, and the apical portion of the parameres is curved (Schuh 2006).

### Distribution and host associations of *Heissophila*

The known distribution of the genus *Heissophila* is significantly expanded by the present study (Fig. 6). *Heissophila macrotheleae* is widely distributed, with records from Thailand (Chiang Rai, Chiang Mai and Sakon Nakhon Provinces), Indonesia (East Kalimantan), and China (Yunnan Province) (Schuh 2006; Yin et al. 2023). Here, we add new provincial records from Guangxi Zhuang Autonomous Region (Chongzuo City, Longzhou County), Hainan Province (Dongfang City) and Fujian Province (Nanping City, Yanping District), based on specimens collected from the webs of unidentified macrothelid spiders in recent years. These localities bridge the previously known Yunnan populations of *Heissophila macrotheleae* and the Fujian-Guangdong populations of the new species, suggesting that the genus may have a more continuous distribution along the southern Chinese coastal and sub-coastal regions than previously appreciated.

Host spider identifications reveal an interesting pattern of host associations. *Heissophila sinica* sp. nov. is recorded from three localities in China: Fuzhou City, Fujian Province; Guangzhou City, Guangdong Province; and Jiangmen City, Guangdong Province. Host spiders collected at these three localities were identified, revealing that the host of *H. sinica* sp. nov. in Fujian is *Orientothele* sp., while in Guangdong it is *Vacrothele* sp. Both spider genera belong to Macrothelidae, the host family of *H. macrotheleae* (associated with *Macrothele* spp.). This pattern indicates that species of *Heissophila* exhibit family-level fidelity to Macrothelidae while showing genus-level plasticity. Such a host strategy may facilitate broader geographic dispersal without requiring strict co-speciation, potentially explaining the relatively wide yet disjunct distribution of the genus.

### Biological notes on *Heissophila sinica* sp. nov.

Field observations at Qishan National Forest Park, Fuzhou, provided detailed ecological insights. Over 50 webs of *Orientothele* sp. were examined on shaded, moist soil slopes. Notably, *H. sinica* was found exclusively on small to medium-sized webs constructed by juvenile spiders; no individuals were detected on large adult webs. On occupied webs, population sizes ranged from 2 to 6 individuals (adults and nymphs) per web, and all field-collected adults were brachypterous.

Laboratory rearing (25 °C, 60% RH, c. 20 adults per web) allowed preliminary life-history observations. Yellow eggs appeared on the webs three days later, with an egg incubation period of about six days; nymphs molted to adults approximately 35 days after hatching. After two generations were reared, one macropterous female adult was found on a web. We hypothesize that the brachypterous condition is better suited for movement within webs, whereas macropterous individuals develop for dispersal when population density on the web becomes excessively high. This hypothesis requires further experimental verification.

## Supplementary Material

XML Treatment for Heissophila

XML Treatment for Heissophila sinica

## Additional information

### Artificial Intelligence (AI) use

The authors accept full responsibility for the content of the manuscript, including the disclosure of any use of AI.

No AI tools were used in the preparation of this manuscript.

### Author ORCIDs

H. Xiong https://orcid.org/0000-0003-1086-5115

Z. Chen https://orcid.org/0000-0003-3393-0338

H. Li https://orcid.org/0000-0001-8590-1753

W. Cai https://orcid.org/0000-0002-8620-0446

### Data availability

All of the data that support the findings of this study are available in the main text.
